# Molecular Basis of NDM-1, a New Antibiotic Resistance Determinant

**DOI:** 10.1371/journal.pone.0023606

**Published:** 2011-08-24

**Authors:** Zhongjie Liang, Lianchun Li, Yuanyuan Wang, Limin Chen, Xiangqian Kong, Yao Hong, Lefu Lan, Mingyue Zheng, Cai Guang-Yang, Hong Liu, Xu Shen, Cheng Luo, Keqin Kathy Li, Kaixian Chen, Hualiang Jiang

**Affiliations:** 1 Drug Discovery and Design Center, State Key Laboratory of Drug Research, Shanghai Institute of Materia Medica, Chinese Academy of Sciences, Shanghai, China; 2 State Key Laboratory of Medical Genomics, Shanghai Institute of Hematology, Rui Jin Hospital Affiliated to Shanghai Jiao Tong University School of Medicine, Shanghai, China; 3 Center for Systems Biology, Soochow University, Jiangsu, China; 4 School of Pharmacy, East China University of Science and Technology, Shanghai, China; Medical College of Georgia, United States of America

## Abstract

The New Delhi Metallo-β-lactamase (NDM-1) was first reported in 2009 in a Swedish patient. A recent study reported that *Klebsiella pneumonia* NDM-1 positive strain or *Escherichia coli* NDM-1 positive strain was highly resistant to all antibiotics tested except tigecycline and colistin. These can no longer be relied on to treat infections and therefore, NDM-1 now becomes potentially a major global health threat.

In this study, we performed modeling studies to obtain its 3D structure and NDM-1/antibiotics complex. It revealed that the hydrolytic mechanisms are highly conserved. In addition, the detailed analysis indicates that the more flexible and hydrophobic loop1, together with the evolution of more positive-charged loop2 leads to NDM-1 positive strain more potent and extensive in antibiotics resistance compared with other MBLs. Furthermore, through biological experiments, we revealed the molecular basis for antibiotics catalysis of NDM-1 on the enzymatic level. We found that NDM-1 enzyme was highly potent to degrade carbapenem antibiotics, while mostly susceptible to tigecycline, which had the ability to slow down the hydrolysis velocity of meropenem by NDM-1. Meanwhile, the mutagenesis experiments, including D124A, C208A, K211A and K211E, which displayed down-regulation on meropenem catalysis, proved the accuracy of our model.

At present, there are no effective antibiotics against NDM-1 positive pathogen. Our study will provide clues to investigate the molecular basis of extended antibiotics resistance of NDM-1 and then accelerate the search for new antibiotics against NDM-1 positive strain in clinical studies.

## Introduction

The New Delhi Metallo-β-lactamase (NDM-1) was first reported in 2009 in a Swedish patient, who travelled to New Delhi and acquired a urinary tract infection caused by *Klebsiella pneumonia* [1]. A recent study reported that *Klebsiella pneumonia* NDM-1 positive strain or *Escherichia coli* NDM-1 positive strain was highly resistant to all antibiotics tested except tigecycline and colistin [2]. Since August 2010, the spreading and dissemination of NDM-1 positive strain has occurred, with cases being globally reported by medias from countries including United States, Canada, Sweden, United Kingdom, Austria, Belgium, France, Netherlands, Germany, Africa, Oman, Australia, Japan and China [3]. Although NDM-1-positive cases are not currently prevalent in the worldwide, it can spread through renal or bone marrow transplantation, dialysis, cerebral infarction, chronic obstructive pulmonary disease, pregnancy, burns, road traffic accidents, and cosmetic surgery. In addition, NDM-1 positive strain can destroy carbapenem antibiotics such as meropenem, imipenem, doripenem and ertapenem by breaking down the carbapenem groups of antibiotics, which have been serving as the basis for the treatment of antibiotic-resistant bacterial infections and now can no longer be relied on for this purpose due to the emergence of NMD-1 positive strain. Therefore, the spread of the pathogenic microorganisms carrying NDM-1 gene (also been called “super bugs”) now becomes potentially a major global health threat.

NDM-1 belongs to the Metallo-β-lactamase (MBL, class B) family containing Zn^2+^ and other divalent cations as cofactors. It inactivates almost all classes of β-lactams antibiotics including carbapenems by catalyzing the hydrolytic cleavage of the substrate amide bond. On the basis of the protein sequence similarities, three different lineages, named as subclass B1, B2 and B3 have been characterized. A number of experimental and theoretical studies have also been devoted to understanding structural and mechanistic properties of MBLs [4], [5], [6], [7]. However, since this novel MBL—NDM-1 is likely more potent and extensive than known MBLs in inactivating β-lactams antibiotics, it is urgent for us to address the ligand binding properties and catalytic mechanism of NDM-1 in order to light up the road for the development of novel antibiotics to combat emerging NDM-1 positive pathogen.

To explore the molecular basis for antibiotics hydrolysis by NDM-1, homology modeling method was performed to obtain the 3D structure. Then molecular docking method was applied to obtain the binding modes with antibiotics and the comparison of NDM-1 with other MBLs complexes was also investigated. In addition, NDM-1 catalyzed hydrolysis of various antibiotics substrates was monitored by following the absorbance variations resulting from the opening of the β-lactam ring, which suggested that NDM-1 displays different resistant abilities to different kinds of antibiotics. Based on the modeling results, four point mutants, including D124A, C208A, K211A and K211E, were made and their enzymatic activities were measured comparing with the wild-type. The significantly decreased activity of mutants validated the accuracy of our model. On the other hand, it shed light on the catalytic mechanism of the novel NDM-1 from the molecular basis for the first time. Furthermore, enzymatic activities toward different antibiotics tested in our study would provide clues to accelerate the new antibiotics design against NDM-1 positive strain in further studies.

## Results and Discussion

### Overall structure of NDM-1 from homology modeling

**Figure 1:**
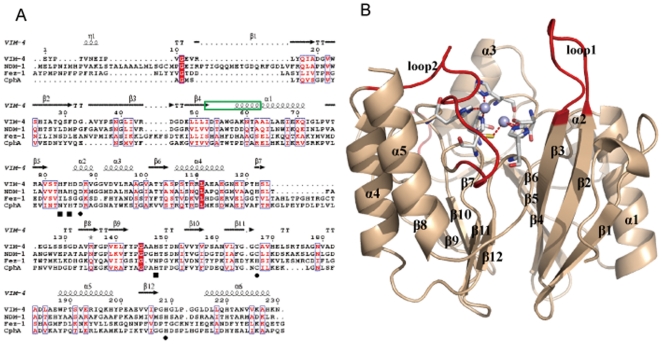
Overall structure of NDM-1 and its sequence alignment with its homologue proteins. A. Sequence alignment of NDM-1 with VIM-4, FEZ-1 and CphA. The second structure assignment of VIM-4 is labeled on the top of the sequences. Black quadrangles indicate residues that coordinate with Zn^2+^(I), while black circles indicate residues that coordinate with Zn^2+^(II). The residues composing of the loop1 of NDM-1 is labeled in green box. B. Cartoon representation of the overall structure of NDM-1 is in light orange color. The loop1, loop2 and the insertion are colored in red, the conserve residues coordinating with zinc ions are represented as sticks in gray color, and the zinc ions shown as spheres.

**Figure 2:**
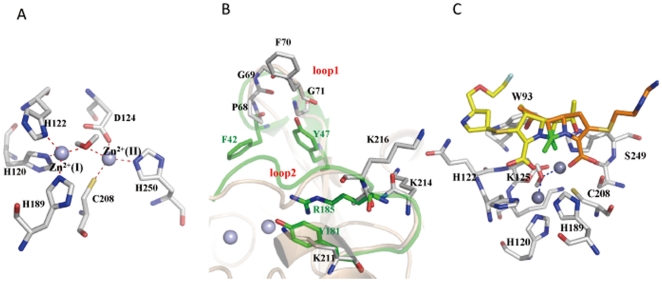
Molecular models of NDM-1 and its complex with antibiotics. A. The active site of NDM-1 with two zinc ions and the coordinating residues. B. The comparison of the two loops in NDM-1 and VIM-2. The loops and key residues in NDM-1 are colored in gray, while in VIM-2 colored in green. C. The binding modes of antibiotics imipenem and carbapenem in the active site of NDM-1. The two antibiotics are colored in orange and yellow sticks respectively, while the key residues in gray sticks. The lactam motifs are all colored in green.

As the multiple sequences alignment shown in Figure 1A, among VIM-4 (B1 subclass), CphA (B2 subclass) and FEZ-1 (B3 subclasses), NDM-1 is highly homologous to the B1 subclass (VIM-4, sequence identity is 37%). In addition, sequence alignment indicates that NDM-1 contains the identical coordinating residues (His-His-His) in the Zn^2+^(I)-binding site and (Asp-Cys-His) in the Zn^2+^(II)-binding site of the B1 subclass, which implies that NDM-1 belongs to the B1 subclass of MBLs. Accordingly, the crystal structure of VIM-4 (PDB ID: 2WHG) was used as the template in homology modeling [8]. As shown in Figure 1B, the overall structure of NDM-1 shares the common characteristic folding of αβ/βα sandwich, which resembles the architectural features in other B1 subclass. The active site of NDM-1 is highly homologous to that of VIM-4. In the active site (Figure 2A), Zn^2+^(I) is coordinated with three conserved histidine residues 120, 122 and 189, while Zn^2+^(II) is coordinated with the conserved residues Asp124, Cys208 and His250, which is exactly consistent with the X-ray crystal structures [9], [10]. A water molecule bridging both zinc ions acts as the nucleophile during the β-lactam hydrolysis.

Two mobile loops in the active site shown in Figure 1B are crucial for substrate recognition, binding and catalysis in MBLs [11], [12]. In NDM-1, the loop1, also called the flapping loop, is composed of amino acids LDMPGFGAVA (residues 65–74). Compared with the loop1 (QSFDGAVYP) in VIM-2 and VIM-4, the size of the active site cavity is enlarged with less bulky amino acids, suggesting a broader substrate profile. Besides it is worth noting that it is more hydrophobic than others [9]. There is only one benzene ring of Phe70 in the middle of the loop1 of NDM-1, and the side-chains of Asp66 and Met67 pointed to the solvent, the less bulky amino acids, especially for the glycines, are proposed to make the loop1 more flexible for substrate binding. In the crystal structure of NDM-1 in complex with hydrolyzed ampicillin (PDB ID: 3Q6X) [9], the loop1 represents a more open state compared with our model, while in the crystal structure of apo NDM-1 (PDB ID: 3S0Z) [10], the loop1 displays a semi-closed state. Consequently, it is proposed to undergo significant conformational changes during the substrate binding, in different states with or without substrate. Regarding the loop2, Arg185 and Asn190 in VIM-2 was proved to play important roles for substrate binding, catalysis and inhibition through H-bond interactions [12], [13]. While in NDM-1, the arginine is mutated to Ala215, which also enlarges the size of active site cavity. Moreover, Lys211 instead of Tyr181 in VIM-2, probably plays the similar role of Arg185 in VIM-2 by forming electrostatic interaction with the carboxyl of substrate. The detailed comparison of the two loops in NDM-1 and VIM-2 is shown in Figure 2B. Furthermore, two residues, Lys125 and Tyr229, were pointed to play crucial roles in stabilizing the conformation of the active site, through H-bond network (with residues Asn76, Asp90, Thr91, His122 and Ser249) and hydrophobic interactions (with residues Leu209, Leu218, Leu221 and Leu269) [9]. Besides the unique residues in the two loops, the N terminus of NDM-1 is longer than other analogues. Even without modeled in our structure, it is speculated to assist in loop1 packing so as to affect the hydrolytic catalysis. NDM-1 also contains an additional insert between residues 162 and 166, which is not present in other MBLs. Since the insert is in the opposite side of the active site, with a distance of around 20 Å, its role in the hydrolysis reaction is still unknown. In summary, the unique structural characteristics probably contributes to the more potent hydrolysis and broader substrate range of the novel MBL—NDM-1, especially for those bulkier antibiotics, which are not degraded by other MBLs.

### Binding modes of antibiotics with NDM-1 suggesting the conserved hydrolysis mechanism

**Figure 3:**
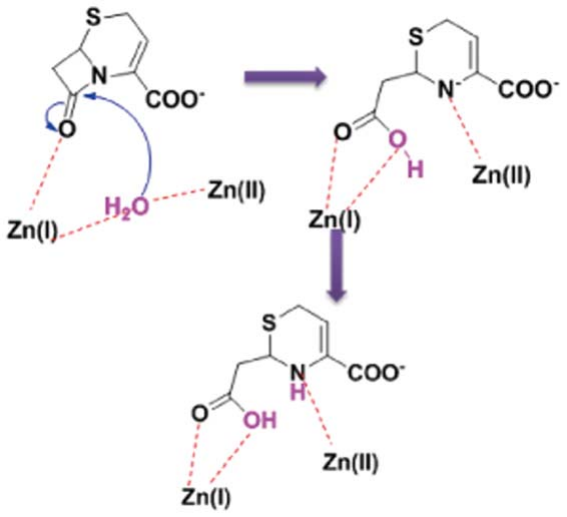
One proposed bis-zinc-form mechanism for the hydrolysis of cephalosporin scaffold by B1 subclass enzyme NDM-1.

After the 3D structure of NDM-1 was modeled, molecular docking study was performed. Two reported effective antibiotics (tigecycline and colistin) against NDM-1 positive strains [2] and other 13 well-known antibiotics including carbapenem, which were reported to be destroyed by NDM-1 positive pathogen, were docked into the active site of NDM-1. The molecular docking data indicates that the latter 13 antibiotics fit the active site of NDM-1 quite well. Taking two typical antibiotics, imipenem and carbapenem as example, the docked complex structures revealed that although the antibiotics adopted diverse conformations in the active site, the lactam motifs were positioned in the same orientation by coordinating with zinc ions tightly (Figure 2C), which suggested that the catalytic mechanisms were highly conserved among B1 subclass enzymes, as shown in Figure 3. Substrate-binding polarizes the lactam bond due to coordination of the carbonyl oxygen with Zn^2+^(I) and the lactam nitrogen with Zn^2+^(II). Attack of the water oxygen leads to oxyanion stabilized by Zn^2+^(I), which is followed by subsequent cleavage of the C-N bond. Then, the lactam nitrogen is expelled as an anion and stabilized by coordination with Zn^2+^(II) acting as a general acid. The last step is the protonation of the nitrogen, which is considered as the rate-limiting and the most controversial step to date [4]. Compare to other known B1 subclass enzymes such as VIM-2 and VIM-4, the loop2 of NDM-1 harbors a lysine-rich positive-charged region, which is more favorable for protonation, probably accounting for its potent catalytic activity at least in part. Notably, colistin, the reported antibiotic susceptible for NDM-1 positive pathogens, was not able to fit the active site well probably due to its bulky volume.

### Comparison the complex structure of NDM-1/meropenem with VIM-2/meropenem and FEZ-1/meropenem

**Figure 4:**
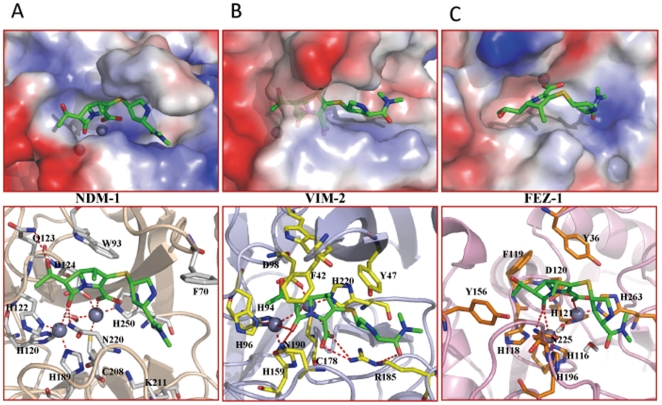
Complex models comparison between NDM-1(A), VIM-2(B) and FEZ-1(C). Upper panel shows the structural electrostatic surfaces (the color blue indicates the positive-charge, while red negative-charged), two zinc ions are shown as gray spheres, and antibiotics meropenem is represented in green sticks. The detailed interactions between meropenem and three enzymes NDM-1 (gray sticks), VIM-2 (yellow sticks) and FEZ-1 (orange sticks) are shown on the lower panel.

**Figure 5:**
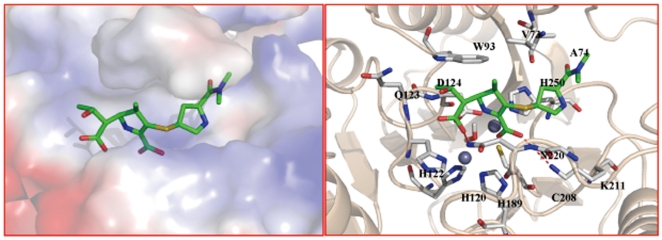
Binding mode of hydrolyzed meropenem with NDM-1. The left displays the structural electrostatic surface and the right is the detailed binding interactions.

To gain the structural insight into the mechanism of the potent hydrolysis of NDM-1, the intermolecular interactions of three models of NDM-1, VIM-2 and FEZ-1 in complex with antibiotics meropenem were compared and analyzed in details (Figure 4A–C). In NDM-1 complex structure, the lactam oxygen and the carboxyl oxygen atoms coordinate with Zn^2+^(I) and Zn^2+^(II) with distances of 3.56 Å and 2.64 Å respectively. In addition, the H-bond between the atom N^Δ2^ of Asn220 and the lactam oxygen atom with distance of 2.96 Å also contributes to the polarization of the lactam bond. Meanwhile, the water molecule, which bridges with the two zinc ions with distances of 1.88 Å and 2.40 Å respectively, can serve as the nucleophile to attack the atom C of β-lactam. The carbonyl methyl of meropenem forms H-bonds with the atom N^ε2^ of Gln123. Together with the fact that the relatively bulky active site cavity caused by the residue variations in the flexible loop1, NDM-1 is potent to hydrolyze bulkier antibiotics. Different with NDM-1/meropenem, the VIM-2/meropenem has a strong H-bond between the carboxyl of meropenem and Arg185 in the loop2. Moreover, Phe61 and Try67 in the loop1 form hydrophobic interactions with meropenem, and dramatically reduce the size of the active site cavity. Structurally, the hydrophobic interactions make the lactam ring rotate to the loop1 and elongate the distance between the lactam oxygen and Zn^2+^(I) to 4.3 Å. (Figure 4B) Consequently, the shallow active site makes the lactam more difficult to be positioned in a proper orientation for catalysis, which probably explains its weaker activity to catalyze antibiotics compared with NDM-1. In contrast, FEZ-1 shares little structural similarity with NDM-1 and VIM-2, especially for that the flapping loop is not conserved in FEZ-1, which leads to a flat active site cleft [14]. Obviously,meropenem mediates no other interactions with FEZ-1 except for the lactam motif (Figure 4C), suggesting the low binding affinity for substrates. And the FEZ-1/meropenem flexible characteristic makes the antibiotic little access to proper orientation for hydrolysis. In agreement with our intermolecular interaction models of enzymes with antibiotics, the reported minimal inhibitory concentrations of meropenem for VIM-2 and FEZ-1 positive *E.coli* strains are both 0.25 µg/ml, while for NDM-1 positive *E.coli* strain, it is 32 µg/ml [2], [15], suggesting that NDM-1 is probably more potent in antibiotics hydrolysis. Besides, the binding modes of hydrolyzed antibiotics were also investigated. Taking the hydrolyzed meropenem for example (Figure 5), it is noticed that the original carboxyl formed electrostatic interactions with Lys211. The hydrophobic residues such as Leu65, Val73 and Ala74 in the loop1 formed stable hydrophobic interactions with the dimethylamino group. It appears that the conformations of antibiotics undergo slight exchange during the catalysis.

### NDM-1 enzymatic activity test prove its potency in the hydrolysis of carbapenem antibiotics

**Figure 6:**
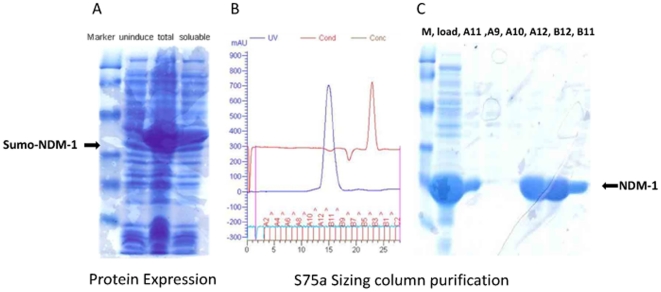
NDM-1 protein expression and purification. A. 6×His and sumo (small ubiquitin-related modifier) tagged NDM-1 was overexpressed in E.coli BL21(DE3) strain, the black arrow indicates the sumo-NDM-1 fusion protein. B. The cleaved NDM-1 protein was purified by S75 gel filtration chromatography. C. SDS-PAGE gel shows the purity of NDM-1 more than 95%.

**Figure 7:**
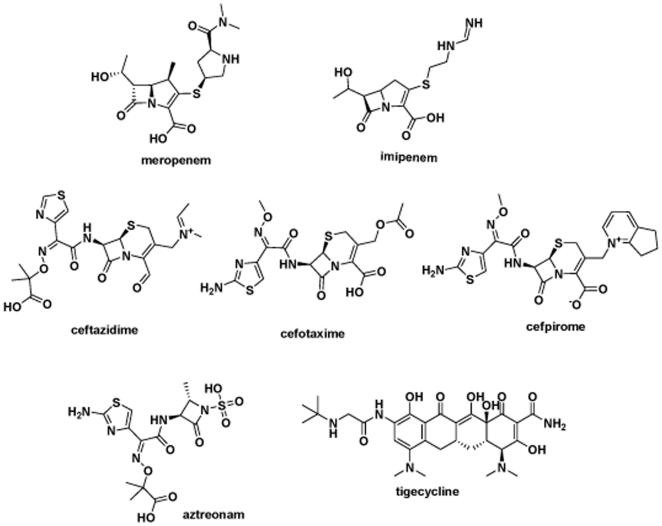
The chemical structures of the seven antibiotics tested in the assay of NDM-1 catalytic activity.

**Figure 8:**
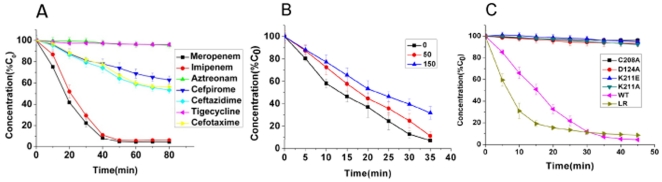
Hydrolysis activity of NDM-1 by enzymatic assays. A. Seven antibiotics were hydrolyzed by NDM-1 protein. The enzymatic reactions were dynamically monitored by the degradation of the antibiotics. C_0_ is the initial concentration of antibiotics. Error bars, s.d. B. Tigecycline inhibited the hydrolysis activity of NDM-1 to meropenem weakly. Compare with control (black line), 50 µM (red line) and 150 µM (blue line) tigecycline slowed down the hydrolysis velocity of meropenem by NDM-1. C_0_ is the initial concentration of meropenem. Error bars, s.d. C. Compare with the wild type enzyme (pink line), the point mutants C208A (black line), D124A (red line), K211A (green line) and K211E (blue line) totally disrupt the hydrolytic activity to meropenem; while mutations of loop1 (dark yellow line) hardly affect the NDM-1 hydrolytic activity. C_0_ is the initial concentration of meropenem. Error bars, s.d.

To validate the results extracted from the *in silico* study, we went ahead to perform “wet” experiments to further explore the detailed molecular basis for the catalytic mechanism of NDM-1. Firstly, we constructed a 6×His and sumo (small ubiquitin-related modifier) tagged NDM-1 expression plasmid and overexpressed sumo-NDM-1 in E.coli BL21(DE3) strain; then we purified fusion protein by Ni-column affinity chromatography, the NDM-1 protein was released from fusion protein by ULP-1 (Ubiquitin-Like protein-specific Protease 1) protease cleavage. More than 95% pure NDM-1 protein was obtained by final gel filtration chromatography (Figure 6A, B and C). Secondly, the NDM-1 catalyzed hydrolysis of various antibiotics substrates was monitored by following the absorbance variations resulting from the opening of the β-lactam ring. The susceptibilities of seven typical antibiotics were assessed. The chemical structures of these antibiotics are displayed in Figure 7. As shown in Figure 8A, NDM-1 rapidly hydrolyzes the carbapenem antibiotics including meropenem and imipenem, and it harbored moderate catalytic hydrolysis ability against cephalosporin antibiotics, including ceftazidime, cefotaxime and cefpirome. In contrast, NDM-1 is susceptible to tigecycline and monobactam—aztreonam, which is similar with the catalytic ability of VIM-4 and NDM-1-positive Enterobacteriaceae [2], [8].

To explore the detailed mechanism for the susceptibility of the two antibiotics against NDM-1 positive strain, 100 µM meropenem was used as NDM-1 substrate, then 50 µM and 150 µM tigecycline or aztreonam were mixed with the reaction system as the inhibitors. Surprisingly, tigecycline probably displayed a certain degree of inhibition against NDM-1. The hydrolysis velocity of meropenem by NDM-1 is partially slowed down when different concentrations of tigecycline were added into the reaction system (Figure 8B). For another NDM-1 positive strain susceptive antibiotic aztreonam, we didn't see any inhibitory effects on NDM-1 favorite substrate (data now shown). In short, our data revealed the molecular basis of the novel MBL-NDM-1, of which NDM-1 positive strain possessed potent resistance to carbapenems. On the other hand, we identified a weak inhibitor—tigecycline, which was previously reported to inhibit the growth of NDM-1 harbored *Klebsiella pneumonia* [2]. Our work may pave the road in designing inhibitors and new antibiotics susceptible against NDM-1.

### Site point mutations of NDM-1 proved Zn^2+^(II) is essential for the hydrolytic activity

Meanwhile, based on our 3D model, we designed the following point mutants, including, D124A, C208A, K211A and K211E. Then the activity of the mutants for meropenem was measured and the result is shown in Figure 8C. It is obvious that the mutants almost completely lose their activity in antibiotics hydrolysis. This result in turn verified the accuracy of our 3D model in which the Zn^2+^(II) is coordinated with the conserved residues Asp124 and Cys208, in spite that it is still intriguing whether the effect is structural or functional. However, the decreased catalytic activity implied the mechanism should be Zn^2+^(II) assisted, supporting the proposal that Zn^2+^(II) ion is crucial for stabilizing the development of a negative charge on the β-lactam nitrogen atom(as show in Figure 3). Regarding K211A and K211E mutants, the significantly decreased hydrolysis activity indicated that the electrostatic interaction between the original carboxyl group of hydrolyzed antibiotics and positive-charged side-chain of K211 probably afford one of the driving force for the catalysis. While with respect to loop1 (LDMPGFGAVA), we did the loop displacement with QSFDGAVYP in VIM-2 and VIM-4. Different with the point mutants, the hydrolytic catalysis didn't decrease compared with the wild-type, which means only the loop1 change wouldn't affect the hydrolytic catalysis very much. Moreover, it was reported that A121F, Q123D and A121F/Q123D mutants strongly weakened the imipenem hydrolysis activity of NDM-1, proving the crucial roles of the unique HA(121)HQ(123)D motif compared with HFHDD motif in other MBLs [10]. Furthermore, since loop1 and loop2 are critical for substrate binding and catalysis, more attention would be paid on the glycines in loop1 and lysines in loop2.

### Conclusions and future prospects

Nowadays, the antibiotics resistance in Gram-negative bacteria has already been a great risk to the public health. The newly emergent NDM-1, called “superbug”, possesses more potent hydrolysis ability toward almost all antibiotics and hence becomes a new threat in clinical surgery. This is one typical example of the remarkable ability of bacteria to adapt and eventually become resistant to new antibiotics. It is made much easier by the existence of plasmids, which can transmit easily from bacterium to bacterium. In this study, the structural models of NDM-1/antibiotics complex were obtained from homology modeling and molecular docking methods. The detailed analysis indicates that the loop1 of more flexibility and hydrophobic, together with the loop2 of more positive-charged, leads to NDM-1 more potent in antibiotics hydrolysis compared with other MBLs. Meanwhile, the experiments proved that NDM-1 was highly resistant to carbapenems and cephalosporins and susceptible to aztreoname and tigecycline, which was firstly implied to slow down the hydrolysis velocity of meropenem by NDM-1 in our study. Moreover, the mutant results displayed the molecular basis for the catalytic mechanism. At present, there are no effective antibiotics against NDM-1 positive pathogen. An appreciated strategy is to identify drug candidates from the existing antibiotics, such as tigecycline, based on the 3D model of NDM-1 by using structure-based virtual screening (e.g., molecular docking and ligand-based, receptor-based and pharmacophore-based drug design) in conjunction with bioassay. This strategy has been used successfully in the discovery of the compound cinanserin against SARS (severe acute respiratory syndrome) [16]. Taken together, our study provided clues to investigate the molecular basis of extended antibiotics resistance of NDM-1 and shed light upon the discovery of new antibiotics against NDM-1 positive strains in clinical studies.

## Materials and Methods

### Homology model procedure

The protein sequence of NDM-1 from Enterococcus faecium (HQ256747) was retrieved from NCBI (http://www.ncbi.nlm.nih.gov) protein database. BLASTP program was then performed to search for its homologues from the RCSB Protein Databank [17]. Accordingly, the crystal structure of VIM-4 (PDB ID: 2WHG) was selected as the template [8], whose sequence identity with NDM-1 is 37%. To analyze the sequence conservation, sequences alignment of NDM-1, VIM-4, CphA and FEZ-1 was performed and gaps were inserted into the sequences to find an optimal alignment as shown in Figure 1. The 3D structure of NDM-1 was then modeled by using the InsightII software (Accelrys Inc., San Diego, CA, USA) and optimized by energy minimization using the Amber force field implemented in the Sybyl software package. It was minimized gradually (hydrogens, side-chains, all) using the constraints in heavy or backbone atoms to alleviate any remaining bad steric contacts. The optimized structure of NDM-1 was then subjected to evaluation by PROCHECK and Profiles-3D to examine the stereochemical quality and the structural rationality [18], [19]. After verified the rationality, the 3D structure of NDM-1 was subjected to the subsequent study.

### Molecular docking method

Glide calculations were performed with Maestro v7.5(Schrodinger, Inc.) [20]. Hydrogen atoms and charges were added during a brief relaxation performed using the Protein Preparation module in Maestro with the “Preparation and refinement” option, and a restrained partial minimization was terminated when the root-mean-square deviation (rmsd) reached a maximum value of 0.3 Å in order to relieve steric clashes. The grid-enclosing box was centered on the Zn^2+^(I) and defined so as to enclose residues located within 10 Å, and a scaling factor of 1.0 was set to van der Waals (VDM) radii of those receptor atoms with the partial atomic charge less than 0.25. In the docking process, extra-precision (XP) docking was adopted to generate the minimized pose, and the Glide scoring function (G-Score) was used to select the final 20 poses for each antibiotic. Together with reported study, the reasonable poses were used for the binding mode analysis.

### Antibiotics

All antibiotics used in this study were purchased from J&K SCIENTIFIC LTD., except for kanamycin purchased from Sangon biotech(Shanghai) Co., Ltd..

### Construction of overexpression plasmid

The NDM-1 gene, which was obtained as a 900 bp PCR product from Huashan hospital. A new PCR product with restriction sites (NdeI on forward site and XhoI on reverse site) encoding truncation protein from amino acid Q37 to the last amino acid (designated as NDM-1_37–270_) of NDM-1 were constructed into a modified pET28 vector. As a result, NDM-1_37–270_ were expressed as fusion protein with a N-terminal 6×His – SUMO (small ubiquitin-related modifier) tag.

### Mutagenesis

The QuickChange II site-directed mutagenesis kit (Stratagene, La Jolla, CA) was used to introduce all the mutations, including D124A,C208A,K211A,K211E, replacing residues of loop1(65–74, LDMPGFGAVA) with VIM-2 corresponding residues (40–48,QSFDGAVYP), into the gene of NDM-1_37–270_.

### Protein Expression and Purification

The expression vectors were transformed into *E.coli* strain BL21(DE3). A 5-ml overnight culture of these transformed bacteria in Luria-Bertani (LB) medium containing 50 µg/ml kanamycin was used to inoculate 1 liter of LB medium containing 50 µg/ml kanamycin and 20 µM ZnCl_2_. Bacteria were cultured at 37°C with shaking, until reach an optical density at 600 nm of 0.6, then transferred to 16°C, induced by 0.4 mM IPTG overnight.

The bacteria were collected by centrifugation and resuspended in 20 ml lysis buffer containing 20 mM Tris, pH 8.0, 200 mM NaCl, 0.1% β-mercaptoethanol per liter culture. The bacteria were ruptured by sonication, and the bacteria debris was removed by centrifugation at 18000 rpm for 30 min. The cleared supernatant was load onto a Ni-column, which pre-equilibrated by lysis buffer, and washed with lysis buffer supplemented with 10 mM imidazole. Then ULP-1 (Ubiquitin-Like protein-specific Protease 1) was add into the Ni-column, digest the fusion protein at 4°C overnight. Collect the flow through, concentrated and load onto a superdex 75 column, which pre-equilibrated by 20 mM Tris, pH 8.0, 150 mM NaCl, 5 mM DTT. Fractions containing NDM-1_37–270_ and corresponding mutation proteins were pooled and concentrated, then stored at −80°C freezer. Protein purity was more than 95%, estimated by sodium dodecyl sulfate (SDS)-polyacrylamide gel electrophoresis. The final proteins concentration were determined by using the molar extinction coefficient at 280 nm of 27,880 M^−1^ cm^−1^.

### Enzyme activity assay

The NDM-1 catalyzed substrate hydrolysation reaction were carried out in UV-Star® 96 well microplates (Greiner Bio-One Ltd.) at room temperature within reaction buffer 50 mM Hepes, pH 7.5. The antibiotics hydrolysation were monitored by Tecan infinite 200 multimode and absorbance microplate readers (Tecan Group Ltd.) in the peak absorbance of various antibiotics.
